# The Benefits and Risks of Consuming Brewed Tea: Beware of Toxic Element Contamination

**DOI:** 10.1155/2013/370460

**Published:** 2013-10-23

**Authors:** Gerry Schwalfenberg, Stephen J. Genuis, Ilia Rodushkin

**Affiliations:** ^1^University of Alberta, Number 301, 9509-156 Street, Edmonton, AB, Canada T5P 4J5; ^2^University of Alberta, 2935-66 Street, Edmonton, AB, Canada T6K 4C1; ^3^Luleå University of Technology, Aurorum 10, 977 75 Luleå, Sweden

## Abstract

*Background*. Increasing concern is evident about contamination of foodstuffs and natural health products. *Methods*. Common off-the-shelf varieties of black, green, white, and oolong teas sold in tea bags were used for analysis in this study. Toxic element testing was performed on 30 different teas by analyzing (i) tea leaves, (ii) tea steeped for 3-4 minutes, and (iii) tea steeped for 15–17 minutes. Results were compared to existing preferred endpoints. *Results*. All brewed teas contained lead with 73% of teas brewed for 3 minutes and 83% brewed for 15 minutes having lead levels considered unsafe for consumption during pregnancy and lactation. Aluminum levels were above recommended guidelines in 20% of brewed teas. No mercury was found at detectable levels in any brewed tea samples. Teas contained several beneficial elements such as magnesium, calcium, potassium, and phosphorus. Of trace minerals, only manganese levels were found to be excessive in some black teas. *Conclusions*. Toxic contamination by heavy metals was found in most of the teas sampled. Some tea samples are considered unsafe. There are no existing guidelines for routine testing or reporting of toxicant levels in “naturally” occurring products. Public health warnings or industry regulation might be indicated to protect consumer safety.

## 1. Introduction

The drinking of tea has a history that likely began in China more than 3000 years ago. It has a relatively recent history in the west beginning in the 16th century when it was introduced to Portuguese priests and merchants. It became popular in Britain in the 17th century. The use of tea bags was not common until after WWII.

Tea originates from the plant *Camellia sinensis*, a tree that may grow up to 52 feet in height unless cultivated. Tea plants require significant rainfall of 50 inches a year and grow in acidic soil. Contaminants may vary in the soil, air, or water in which the plants are grown. Acidic soil may result in excess available aluminum and fluoride [1]. An acid or alkali soil pH also enhances leaching of toxic heavy metals from the soil [2]. Increasing pH with soluble calcium would reduce the absorption of fluoride [1]. Environmental pollutants such as fluoride and aluminum have been found in tea in part due to the tea plants absorption and deposition and concentration of these compounds in the leaves [3]. The drinking of more than 5 liters of tea per week may result in dental or skeletal fluorosis [4]. Mercury, lead, arsenic, and cadmium as well as other toxic elements have been found in tea leaves as described in the literature [5, 6]. Lead, arsenic, and cadmium have also been found in brewed black tea [7]. These soil and air contaminants may be directly related to the use of coal fired power plants. The use of coal in China has increased to 3.8 billion tons or about 47% of global coal consumption. Coal burning power plants supply 70% of the energy in China [8]. Pollutants such as lead and mercury from power plants are affecting the development of children, with lead resulting in significant decrease in social and average developmental quotients [9].

Teas are commonly grouped into 5 major categories: white, yellow, green, oolong, and black tea. All of these are readily available at most supermarkets in Canada except yellow tea. For the purposes of this study common off the shelf teas either organic or regular (not labeled as organic) were obtained as well as some that were available in health food stores. All teas used were in tea bags used for brewing in individual cups. The four types of tea sampled in this study are white, green, oolong, and black tea.

Processing of different types of teas is as follows.

1. White tea: young leaves or new growth buds, withered, uncured, baked dry;
2. Green tea: steamed or dry cooking in hot pans to prevent oxidation; dried tea leaves may be separate leaves or rolled into pellets (gunpowder tea);
3. Oolong tea: withering of leaves under sun and warm winds with further oxidation standard between green and black teas;
4. Black tea: leaves are completely oxidized, withered, and disrupted or macerated to activate oxidation resulting in catechins being transformed to complex tannins.

This study will look at some of the benefits of tea as well as the toxicants found in tea. The possible beneficial and medicinal aspects of tea as well as the detrimental effects of heavy metals in tea are discussed below.

## 2. Medicinal Value of Green Tea

Green tea provides a small amount of magnesium, calcium, potassium, phosphorus, and other trace elements considered necessary for health. The results in our study are reported below. Tea contains catechins, which are a type of antioxidant. White and green teas have the highest concentration of these while oolong and black teas have less due to the oxidative preparation. Tea also contains caffeine which may vary from 30 to 90 mg/cup depending on the type of tea and method of brewing. Other medicinal ingredients are theobromine and theophylline found in smaller quantities. There are many and varied effects of drinking tea which are outlined below.

### 2.1. Cardiovascular Effects

Many reports in the literature suggest benefit to the cardiovascular system by reducing cholesterol, reducing coronary artery disease, ameliorating hypertension, and inflammation. Green tea has been shown to reduce total and LDL cholesterol significantly as shown in a recent meta-analysis [10]. Total cholesterol was reduced by 7.20 mg/dL and LDL by 2.19 mg/dL. A dose response curve has been observed. Black tea has not been associated with decreased risk of coronary artery disease, as outlined in a recent meta-analysis, but there may be some benefit from green tea with a 28% risk reduction. However, more robust studies are needed [11]. As well green tea may have some antithrombotic effects [12, 13]. A randomized control study in obese hypertensive patients showed that green tea extract may significantly reduce hypertension (both systolic and diastolic BP), insulin resistance, total and LDL cholesterol, inflammation, and levels of markers of oxidative stress [14].

### 2.2. Anticancer Effects

A Cochrane review (2009) found conflicting evidence that green tea drinking prevents cancer [15]. More recent studies again show conflicting results with benefit for some cancers but not for others. Green tea may reduce risk in developing breast cancer. This effect has been ascribed mostly to the phytochemicals which can modify the metabolism of estrogens [16]. These include polyphenols (catechins) such as epigallocatechin gallate (EGCG), which appears to be most potent, epigallocatechin, epicatechin gallate, and epicatechin. Green tea drinking does not appear to reduce the risk of developing prostate cancer and black tea may increase prostate cancer risk [17]. A most recent meta-analysis shows that the consumption of green tea and coffee appears to reduce esophageal cancer but black tea does not [18].

### 2.3. Weight Loss and Diabetes

A Cochrane review on green tea preparations [19] and weight loss showed a small nonsignificant loss of weight in obese or overweight adults likely not of clinical relevance.

In regard to diabetes green tea may result in lower fasting glucose levels but no significant HbA1c changes [20]. There is some evidence that insulin resistance may be improved by the antioxidant protective function of the polyphenols [21]. As well there is evidence that these antioxidants may protect the retina from neurodegenerative changes seen in diabetic retinopathy [22] and protect against nephropathy [23]. 

### 2.4. Anti-Infective Properties

 Green tea extract potentially could be used in a mouth wash as a preventative for tooth decay and periodontal disease because of its strong antibacterial properties [24]. Epigallocatechin gallate, the most active component of green tea, has antiviral, antibacterial, and antifungal properties [25]. 

### 2.5. Miscellaneous Effects

Tea may reduce mercury absorption [26, 27] and provide protection of nephrotoxicity [28–30]. 

The consumption of greater quantities of tea, 4 or more cups compared to 1 or less, may provide some protection from depression [31]. Another study in elderly patients suggests the same [32].

There is some evidence that the polyphenols in green tea may be protective against Alzheimer's; however further studies are required [33]. 

## 3. Detrimental Effects of Heavy Metals in Tea

There is an abundance of literature demonstrating the adverse health effects of various heavy metal and metalloid elements on the human organism. By numerous mechanisms, including endocrine disruption [34], cytotoxicity [35], mitochondrial dysfunction [36], and oxidative stress [37, 38], a spectrum of toxic elements is able to disturb cellular and metabolic homeostasis and induce clinical illness. The literature is replete with many common disease processes such as carcinogenesis [39], insulin resistance [40], neurodegeneration [41], and immune dysregulation [42, 43]. These may result from exposure to and bioaccumulation of various heavy metals and metalloids. In addition, recent literature has elucidated that various toxic compounds can have epigenetic effects with the potential for transgenerational damage [44, 45]. Rather than isolated incidents of single exposures, it is apparent that toxic metal contact is a widespread phenomenon [46] with many potential sources including tainted food and drink, contaminated skin products, and contaminated air. Many toxic metals such as cadmium and lead have very long half-lives and thus are classified as persistent toxicants [47]. As some toxic elements appear to persist because of enterohepatic recycling [48, 49], even smaller levels of exposure can bioaccumulate and effect long-term harm.

 The toxic elements discussed in this paper include lead, mercury, aluminum, and cadmium. The extremely low levels of lead accepted in Proposition  65 during the prenatal period come from our knowledge of the accumulation in the brain and resultant impairment of cognitive development [9, 50].

## 4. Methods

Distilled water was analyzed after boiling in Pyrex and then allowed to stand in fine bone china cup for 3 minutes. The steeping of the teas was done in the fine china cups as might be done in the real world.

All tea leaves were analyzed to determine the presence or absence of metals.

All teas were steeped using one tea bag (containing 2-3 gm of tea) in 250 mL of distilled water in fine bone china cups. All teas had two samples taken, one steeped for 3-4 minutes and another steeped for 15–17 minutes.

### 4.1. Sample Preparation for Analysis

Water: samples were diluted 10-fold with 1.4 M HNO_3_ (SP grade).

Liquids (waters and brewed tea): samples were diluted 10-fold with 1.4 M HNO_3_ (SP grade).

Solids: approximately 0.25 g of sample was subjected to closed-vessel MW-assisted digestion (MARS-5 oven, 600 W 1 h holding time) using 5 mL concentrated HNO_3_ (SP grade), 0.5 mL H_2_O_2_ (PA grade) and 0.02 mL HF (SP grade). After digestion, solutions were diluted with 1.4 M HNO_3_ (SP grade) providing final dilution factor of approximately 500. A set of digestion blanks and matrix-matched CRM were prepared together with each digestion batch.

All solutions were spiked within (internal standard, at 2 *μ*g/L) and analyzed by ICP-SFMS (ELEMENT2, ThermoScientific) using combination of internal standardization and external calibration.

This analytical method is simple, efficient, and environmentally friendly. The results in this study are consistent with those found in other studies [7].

## 5. Results

### 5.1. Minerals Found in Tea

In our study 4 cups of tea may supply as much as 1% of daily calcium requirements, 5% of magnesium requirements, 4-5% of daily potassium requirements and 1–1.6% of phosphorus requirements using distilled water. See Table 1. The use of regular tap water may provide more magnesium, calcium potassium, and phosphorus than using distilled water.

Trace minerals found in the brewed tea samples were boron 19–115 *μ*gm/L, cobalt 0.4–3.56 *μ*gm/L, copper 26–106 *μ*gm/L, chromium 0.2–14.6 *μ*gm/L, iron 19–62.5 *μ*gm/L, manganese 534–6351 *μ*gm/L, molybdenum 0.03–0.131 *μ*gm/L, phosphorus 3172–20286 *μ*gm/L, selenium <0.1–0.34 *μ*gm/L, vanadium <0.01–0.151 *μ*gm/L, and zinc 44.6–187 *μ*gm/L.

### 5.2. Heavy Metals in Tea

There are established upper limits of ingestion on a daily basis of heavy metals from various organizations. The most stringent are from Proposition  65 in California. 

These limits have been outlined in Table 2 along with other accepted limits.

The levels of toxic elements in this study for mercury (Hg), lead (Pb), aluminum (Al), arsenic (As), and cadmium (Cd) are outlined in Table 3.

Of the 30 teas tested none had detectable levels of mercury as brewed teas although 18/30 tea leaves had detectable mercury present (as high as 20 ng/g of tea). It appears that the mercury is bound in the leaf in a way that it does not make its way into the brewed tea at levels that are detectable (detectable limit in this assay is 0.005 *μ*gm/L).

All teas contained significant amounts of aluminum. Tea leaves contained from 568 to 3287 ng/g of tea. All brewed teas steeped for 3 or 15 minutes contained detectable levels of aluminum. The range was 1131 *μ*gm/L to 8324 *μ*gm/L steeping for 3 minute and 1413 *μ*gm/L to 11449 *μ*gm/L steeping for 15 minutes. Only 2 teas had levels above acceptable limits at 3 minutes of brewing but 6 of the teas had levels greater than the upper acceptable daily limit of 7000 *μ*gm/L. Clearly letting tea steep for longer than 3 minutes is not advisable. Two of the organic green teas had levels above 10,000 *μ*gm/L brewed for 15 minutes.

All brewed tea and tea leaves had detectable lead levels with Chinese oolong teas having the highest levels, followed by green tea and regular black tea having lower levels. Organic white teas had the lowest lead level. Levels ranged from 0.1 *μ*gm/L to 4.39 *μ*gm/L after subtracting the level found after brewing distilled water in fine china cups. 

All brewed tea and tea leaves had detectable arsenic with Chinese oolong teas (organic or regular) having the highest levels. Levels in all teas ranged from 0.06 *μ*gm to 1.12 *μ*gm/L of tea steeped for 3 minutes to 0.08 to 1.27 *μ*gm/L of tea steeped for 15 minutes.

All tea leaves had detectable levels of cadmium. 21 teas had detectable levels after 15 minutes brewing while only 18 teas had detectable levels after 3 minutes brewing suggesting that there is further leaching of this toxicant into the water over time. The highest level was 0.067 *μ*gm/L found in standard oolong tea from China.

All tea leaves and brewed teas had detectable levels of cesium with one organic tea having 3103 ng/g in the dry leaf, 12.4 *μ*gm/L at 3 minutes of brewing and 16.5 *μ*gm/L at 15 minutes of brewing.

All tea leaves had detectable levels of tin but only two brewed samples had nonsignificant levels detected in the teas.

All tea leaves and all teas had detectable levels of barium, antimony and thallium but none had levels considered to be of concern.

## 6. Discussion

Heavy metal contamination in tea has been described in the literature before using the same method of analysis [7]. Lead levels in the previous study were found to be the highest in Chinese samples as seen in our study. Infusion for 15 minutes increased the amount of toxicants in the previous study similar to this study.

The benefits of green tea as outlined above are multiple, and tea may contribute to the daily intake of essential minerals and benefit overall health. However, in the real world of tea drinking it is important to look at several other factors to minimize exposure to heavy metals.

First the source of tea and where it was grown (country of origin) must be considered; see Table 4. It would be optimal to drink tea with minimal exposure to toxicants in ground water, soil, air, and rain. Second one must consider the water that the tea is brewed with that may contain contaminants. Tap water does contain more contaminants than distilled water. Third the vessels that the water is boiled in may contribute to toxicants and the cups either glass or fine china used for steeping may or may not contribute to the toxic load. In this study the leaching of lead from the fine china cups into distilled water alone resulted in a lead level of 0.4 *μ*gm/L. This was subtracted from all the analysis results of the teas to obtain the true level contributed by the brewed tea.

Of the trace minerals manganese is the only mineral found in substantial amounts in teas and some teas will supply more than the total daily requirements. Black tea achieved the highest level in this study. Excess manganese can result in interference with the absorption of iron [52] and may result in ADHD-like symptoms in children exposed in utero [53].

In regard to toxic elements tested only aluminum and lead had levels that were unacceptable. Unacceptable aluminum levels were found in 7% of teas brewed for 3 minutes and 20% of teas brewed for 15 minutes. The lead levels that were present are a significant concern during pregnancy in that 73% of the samples brewed for 3 minutes and 83% of samples brewed for 15 minutes were above 0.5 *μ*gm/L. Despite smaller amounts of cadmium and arsenic there is concern for long-term bioaccumulation.

The organic teas had significantly higher levels of lead contamination if left steeping for more than 15 minutes than the regular teas. Otherwise there was no significant difference in levels of contaminants between organic teas and regular off the shelf teas. Organic teas did not appear to have less toxic element contamination than regular teas even from the same company.

## 7. Limitations of the Study

 A limitation of this study is that this is a sample of convenience using samples readily available in supermarkets and health food stores in Canada. This study did not look at fluoride, which is a very common and significant contaminant. The particular method of analysis used here would not allow for fluoride analysis. The scope of this analysis did not look at pesticides, herbicides, or other organic contaminants, which are addressed in some of the organic labeling.

## 8. Recommendations Associated with This Study

 Although manganese is an essential trace mineral, levels in black tea are quite high and may result in toxic levels when adjusted for total daily intake from multiple sources. 

The acceptable limit of lead in reproductive health is 0.5 *μ*gm on a daily basis. All but 5 teas or 83% of teas had levels above this limit when consuming 4 cups of tea daily. Consumption of tea needs to be severely limited during pregnancy. The consumption of this and some prenatal vitamins [51] may easily exceed this daily limit and result in significant bioaccumulation over time especially in the fetus. As well when the additional lead from the tea cup was added, 100% of all teas had more than the acceptable limit of lead.

The allowable limit for lead ingestion for adults is 15 *μ*gm daily. All brewed teas had detectable levels of lead above that found using distilled water in fine china tea cups and one of the teas had 4.4 *μ*gm/L of lead. Since tea is only part of what may be ingested on a daily basis this may be significant. Some nutritional supplements also have high levels of lead especially Chinese and Ayurvedic herbal remedies [51]. In combination it would be easy to exceed this daily limit. Chinese oolong teas had the highest levels of lead and although this is below the acceptable standard of 15 *μ*gm/day are best avoided.

All teas had significant levels of aluminum and 6 out of 30 teas brewed for 15 minutes had unacceptably high levels. Drinking more than 4 cups of tea a day may contribute significantly to a toxic load.

Brewed tea appears to contain numerous toxic elements such as arsenic and cadmium. However, none of these toxicant levels were above present day acceptable standards. 

Steeping tea for longer periods of time increases the levels of these contaminants by 10 to 50% over steeping for 3 minutes. Therefore steeping for longer than 3 minutes should be avoided.

Although mercury is found in the tea leaves no mercury was detected in the brewed tea even when steeped for longer periods of time. This raises an interesting question: would tea be useful for detoxification from mercury? 

The source of water used for brewing may contain some contaminants and add to the toxic load.

One must know the manufacturing source and processing of the cups in which the tea is brewed especially fine china cups that may contain lead in the glaze. There are manufactures that advertise having no lead glaze and glassware is unlikely to have lead.

## 9. Conclusions

To move forward in diminishing the risk of toxic element contamination a few points are offered for consideration. When determining regulatory standards as well as individual and public health recommendations, it is important to consider the cumulative total load of toxic elements as some of these agents including cadmium and lead have long half-lives [47] and constitute persistent pollutants within the human body. As such, it is important to consider means to diminish exposure as well as to facilitate elimination of toxic elements from all sources, including beverages. Precautionary avoidance is paramount as individuals and public officials should consider mechanisms to limit exposures that add to the total burden. It is insufficient to simply look at isolated exposures to toxic elements, but rather to look at the total cumulative exposure. 

As such, if individuals are being exposed to lead, for example, from drinking polluted tea as in this study, from taking tainted supplements [51], from consuming contaminated drinking water from lead pipes [54], from eating or drinking from dinnerware containing lead [55], and so on, the total daily exposure may be enormous. Simple regulation to control the exposure from one source is insufficient to secure safety from toxic element bioaccumulation and thus education in precautionary avoidance from all main sources needs to be implemented. 

Education to medical trainees about exposures to toxic elements and persistent organic pollutants has been limited in most medical centers thus far. Recognizing the escalating problem of toxicant bioaccumulation [56], it would be prudent to commence education of health professionals, inline with recommendations from the World Health Organization [57] and other notable health bodies.

Public awareness campaigns may be effective in alerting individuals to concerns related to toxic element bioaccumulation and potential sources of exposure. Such awareness may facilitate further avoidance as well as medical intervention to eliminate accrued toxicants [48].

In response to the Pediatric Academic Societies admonition that “low level exposure to environmental toxicity may be impacting the functioning of the current generation,” [58] education programs in schools may have some role in protecting and guiding developing children and their families. 

Routine inspection and testing of foodstuffs and beverages to rule out contamination—with results being made generally available—might identify compounds that are heavily tainted and thus preclude contaminants from being consumed. This might include a self-regulatory process that is overseen by government officials.

Original source labeling of products would provide consumers with information about the geographic origins of products. As some jurisdictions appear to have a greater problem with contamination [51], this will give consumers choice in their acquisitions and the opportunity to provide feedback to jurisdictions which consistently demonstrate contamination.

## Figures and Tables

**Table 1:** Healthy minerals found in teas in this study mg/L.

Calcium		Daily minimum requirement 800 mg
3-4 minute steeping	1.2–8.6	
15–17 minute steeping	1.8–12.8	
Magnesium		Daily requirement 250–300 mg
3-4 minute steeping	2.6–14.4	
15–17 minute steeping	3.4–16.8	
Potassium		Daily requirements 4500–5000 mg
3-4 minute steeping	55–201	
15–17 minute steeping	94–216	
Phosphorus		Daily requirement of 1250 mg
3-4 minute steeping	3.1–17.9	
15–17 minute steeping	4.2–20.3	

**Table 2:** Established toxicant limits in supplements (*µ*gm/day) adapted from [51].

Toxic element	US California Proposition 65 (P65) and Environmental Protection Agency	European union	Australia	World Health Organization	Gestational limits
Mercury (Hg)	2	4.2	2.4 inorganic Hg 0.96 methyl Hg	1.37 (methyl Hg in children)	0.6 for methyl Hg
Lead	15	21	NE	21	Concern at low levels. 0.5 established for reproductive toxicity (P65)
Cadmium	4.1	6	15	6	NE
Arsenic	10	13.0	NE	12.85	NE
Aluminum	7,000	4,286	12,000	NE	NE

NE: not established.

European/WHO/Australian levels were established by convention as representing 10% of the daily total toxicant intake after conversion of values expressed in mg/kg/week for an average adult weight of 60 kg.

**Table 3:** Toxic element contaminants in teas.

TEAS	Steeped 3-4 minutes					Steeped 15–17 minutes				
Average *μ*g/L	Hg	Pb	Al	As	Cd	Hg	Pb	Al	As	Cd
Organic green *N* = 10	None detected	1.64 SD ± 0.58 MX 2.57	3897 SD ± 2027 MX 8324	0.31 SD ± 0.09 MX 0.44	0.021 SD ± 0.01 MX .036	None detected	2.147 SD ± 0.58 MX 3.11	5210 SD ± 2360 MX 10100	0.39 SD ± 0.14 MX 0.68	0.031 SD ± 0.015 MX 0.068

Regular green *N* = 7	None detected	1.37 SD ± 0.47 MX 1.89	2342 SD ± 1401 MX 5635	0.43 SD ± 0.18 MX 0.62	0.012 SD ± 0.008 MX 0.027	None detected	1.51 SD ± 0.55 MX 2.55	4798 SD ± 1970 MX 7417	0.4 SD ± 0.23 MX 0.82	0.02 SD ± 0.012 MX 0.044

Organic oolong *N* = 2	None detected	1.96	5624 MX 5939	0.60	0.01	None detected	2.26 MX 3.12	8808 MX 11449	0.885	0.014

Standard oolong *N* = 2	None detected	3.05 MX 4.13	4150	0.88	0.06	None detected	3.45 MX 4.93	2876	0.95	0.065

Organic white *N* = 3	None detected	1.14 MX 1.95	2875	0.45	0.02	None detected	1.32	4078	0.56	0.03

Organic black *N* = 1	None detected	0.78	5341	014	None detected	None detected	0.79	8096	0.23	None detected

Standard black *N* = 5	None detected	0.86 MX 1.03	4503 MX 8076	0.336	0.026	None detected	0.93 MX 1.33	5261.4 MX 8987	0.28	0.02

SD is standard of deviation. None detected is below the limit of detection or <0.005 ng/L and MX is maximum level detected.

**Table 4:** Toxicant (heavy metal) levels according to country of origin.

Tea type	Tea country of origin	High levels	Moderate levels	Low levels
Green tea organic	China	Pb, Al	As	Cd
Green tea organic	Sri Lanka			Low in Pb, Cd, Al, As
Green tea organic	Japan		As, Pb, Cd, Al	
Green tea standard	China	High in Al	Pb, As	Low in Cd
White tea	India			Lowest in Al, Pb, Cd, As
White tea	China		Pb, Al, As	Low in Cd
Oolong tea organic	China	Highest in As, high in Pb, Al		Low in Cd
Oolong tea standard	China	Highest in Pb	Al, Cd, As	
Black tea organic	Blend India Sri Lanka	Al	Pb	Low in Cd, As
Black tea standard	India Sri Lanka	Al		Lowest Pb, low in Cd, As

Pb: lead, Cd: cadmium, Al: aluminum, As: arsenic.
